# Karyological differentiation among bread wheat cultivars (Triticum aestivum L.) with distinct breeding statuses and growth habits

**DOI:** 10.18699/vjgb-25-83

**Published:** 2025-10

**Authors:** A.F. Muterko, E.D. Badaeva, E.V. Zuev, E.A. Salina

**Affiliations:** Institute of Cytology and Genetics of the Siberian Branch of the Russian Academy of Sciences, Novosibirsk, Russia; Vavilov Institute of General Genetics of the Russian Academy of Sciences, Moscow, Russia; Federal Research Center the N.I. Vavilov All-Russian Institute of Plant Genetic Resources (VIR), St. Petersburg, Russia; Institute of Cytology and Genetics of the Siberian Branch of the Russian Academy of Sciences, Novosibirsk, Russia

**Keywords:** common winter and spring wheat, modern and local cultivars, landrace, breeding, karyological analysis, karyosystematics, C-banding, мягкая яровая и озимая пшеница, староместные и современные сорта, селекция, кариологический анализ, кариосистематика, С-окраска хромосом

## Abstract

The assessment of intraspecific variability of wheat has been relevant for years. Although most modern wheat cultivars are considered to be pure lines, the heterogeneity of varietal populations is one of the mechanisms for maintaining population homeostasis. It is possible that the high evolutionary stability of constitutive heterochromatin and its stable distribution within chromosomes will allow us to use karyological analysis not only for studying the genesis and taxonomy of Triticum L., but also for studying the intraspecific diversity of wheat. In this regard, a classification of 87 Russian cultivars of common wheat differing in breeding status (landraces and modern cultivars) and growth habit (spring and winter) was carried out using two alternative approaches for assessing karyograms. The first approach uses the qualitative assessment of karyograms based on the distribution of C-bands on chromosomes. We also proposed that quantification of karyograms based on the size of C-bands would make the classification of cultivars more adequate. The variability, informative value and resolution of diagnostic features, trends in grouping cultivars, and their associations with the breeding status and growth habit were studied. A high potential of karyotyping with C-banding in discriminating modern cultivars by growth habit, as well as in separating winter cultivars from landraces has been revealed. In terms of the tested karyological features, the homogeneity of modern cultivars was higher than that of local cultivars, and the homogeneity of winter wheat was higher than that of spring wheat. The obtained classification reflects the preservation of high similarity in the karyograms of modern spring cultivars and landraces, as well as the low distinguishability between the karyograms of landraces differing in growth habit. A comparative analysis of the classifications of 20 cultivars using C-banding and SNP genotyping (3,126 polymorphic markers) suggests that studying the karyotypic variability allows us to infer a more accurate differentiation of wheat varietal populations based on the breeding status than using SNP markers that detect genetic variability, especially when the number of diagnostic features is limited.

## Abstract (ru)

На протяжении многих лет оценка внутривидовой изменчивости пшеницы не теряет своей актуальности. Хотя большинство современных сортов пшеницы относят к чистолинейным, гетерогенность сортовых популяций выступает одним из механизмов поддержания популяционного гомеостаза. Возможно, высокая эволюционная стабильность конститутивного гетерохроматина и его устойчивое распределение на хромосомах позволят эффективно использовать кариологический анализ не только для исследования генезиса и таксономии рода Triticum L., но и для изучения внутривидового разнообразия пшеницы. В этой связи проведена классификация 87 российских сортов мягкой пшеницы различного селекционного статуса (староместные и современные сорта) и типа развития (яровые и озимые) на основании оценки кариограмм, выполненной с использованием двух подходов. Первый подход основан на качественной оценке кариограмм по распределению гетерохроматиновых С-блоков на хромосомах. Мы также предположили, что количественная оценка кариограмм по размеру индивидуальных С-блоков (второй подход) сделает классификацию сортов более адекватной. Исследовались вариабельность, информативность и разрешающая способность диагностических признаков, тенденции в группировании сортов, а также их ассоциации с селекционным статусом и типом развития. Результаты выявили высокий потенциал С-окраски в дискриминации современных сортов мягкой пшеницы по типу развития и обособлении их озимых форм от местных культур. Гомогенность современных сортов по тестируемым кариологическим признакам была выше, чем староместных, а озимых – чем яровых. Полученная классификация отражает сохранение высокой общности в кариограммах современных яровых культур и сортовых популяций местного возделывания, а также слабую различимость кариограмм староместных сортов, контрастных по типу развития. Сравнительный анализ классификаций 20 выборочных сортов по данным С-окрашивания и ОНП-генотипирования (3126 полиморфных маркеров) предполагает, что изучение кариотипической изменчивости помогает составить более верное представление о дифференциации сортовых совокупностей пшеницы по селекционному статусу, чем при использовании ОНП-маркеров, детектирующих генную изменчивость, особенно при ограниченном количестве диагностических признаков.

## Introduction

Cultivation of common wheat Triticum aestivum L. (2n = 6x = 42, BBAADD) in various eco-geographical regions with its exceptionally wide distribution has led to the accumulation of structural, genetic and physiological changes, and the emergence of a huge diversity of intraspecific forms (Zohary et al., 2012; Zhao et al., 2023). In this regard, the study of wheat intraspecific variability seems relevant. Although most modern wheat cultivars are classified as pure lines, their inherent polymorphism, heterogeneity of varietal populations, acts as one of the mechanisms for maintaining population homeostasis (Fadeeva, Narbut, 1969; Kudriavtsev, 2006; Serpolay-Besson et al., 2011), which leads to poorly reproducible results in their differentiation (Kudriavtsev, 2006; Metakovsky et al., 2024). The high evolutionary stability of heterochromatic blocks, as well as their stable distribution on chromosomes, creates the prerequisites for the successful use of chromosomal markers to solve such problems.

Previously, karyological analysis has proven itself successful in the study of the genesis and taxonomy of wheat within the genus (Iordansky et al., 1978a, b; Zurabishvili et al., 1978; Badaeva et al., 1986, 1994, 2007, 2015a, 2022; Gill et al., 1991; Jiang J. et al., 1993, 1994; Friebe, Gill, 1996; Dedkova et al., 2004, 2007, 2009); however, the validity and prospects of its application in assessing the population variability of this crop remain a subject of discussion. This is largely due to both the relatively high labor intensity of karyological analysis and the complexity of describing the karyotype of a variety in a form accessible for statistical processing. So, the karyogram of the model variety Chinese Spring serves as a standard for describing deletion lines (Endo, Gill, 1996) and compiling physical maps of chromosomes (Delaney et al., 1995; Mickelson-Young et al., 1995), but due to the absence of a number of C-bands present in other species or cultivars of wheat, it cannot be directly used to characterize intraspecific polymorphism. At the same time, although many authors have noted a wide variety of differential staining patterns of common wheat chromosomes (Iordansky et al., 1978a; Zurabishvili et al., 1978; Seal, 1982; Friebe, Gill, 1994), the karyograms they provide are not suitable for statistical processing and analysis of population structure

Improvement of the fluorescent in situ hybridization method and the development of an oligoprobe system have significantly simplified and reduced the cost of the analysis and made it possible to study fairly large samples (Jiang M. et al., 2017; Huang et al., 2018; Guo et al., 2019; Hu et al., 2022). At the same time, the assessment of the frequency and distribution of polymorphic variants served to identify groups of closely related cultivars (Huang et al., 2018; Guo et al., 2019; Hu et al., 2022). Another approach, “chromosomal passportization”, is based on a comparison of the karyotype of a specific sample with a generalized species ideogram (Badaeva et al., 1990). In contrast to the previously discussed approach, the diagnostic feature here is an individual block of constitutive heterochromatin (C-band). This approach was first used to assess the diversity of spelt wheat (Dedkova et al., 2004) and the European emmer group (Dedkova et al., 2009), and the population structure of T. dicoccum obtained with its use corresponded well to the existing taxonomy (Goncharov, 2012; Badaeva et al., 2015b). The population structure of T. araraticum, revealed using chromosomal analysis (Badaeva et al., 2022), was completely consistent with the data of a molecular genetic study of the same samples, performed using SSAP markers.

Depending on the breeding status, a distinction is made between landraces (local populations) and modern wheat cultivars. Local varietal populations are locally adapted, they are traditionally cultivated in isolated areas, and their seed production is carried out without deliberate hybridization and targeted change of genotype (Zeven, 1998). Nevertheless, the genetic diversity of landraces was maintained by cultivating a mixture of different genotypes or often even a mixture of different crops, which created the possibility of exchanging genetic material between plants (Zeven, 1980, 1998; Feldman, 2001). The transition to scientifically based breeding of common wheat, based on targeted selection of parental genotypes, introgression of foreign genes, and the use of mutagenesis took place in the late 19th–early 20th centuries (Feldman, 2001). Since samples from geographically separated populations, as well as representatives of related taxa, were often used in the creation of cultivars (Mujeeb- Kazi et al., 2013; Sharma M. et al., 2020; Sharma S. et al., 2021; Boehm, Cai, 2024), the possibility of the formation of qualitatively new, specific karyotypes, isolated and maintained in the pool of breeding cultivars, cannot be ruled out. This circumstance actualizes the assessment of the potential of karyological analysis in the differentiation and classification of wheat varietal populations

While the relationship between the karyotype of a given cultivar and its origin has natural grounds, it is fundamentally important to test the associations of the karyotype with other, less obvious – but significant in its formation – factors. One of these factors is the climate regime of the cultivation region, since it has a direct impact on the formation of the growth habit. In particular, winter cultivars are sown before the onset of cold winter weather, supporting the vernalization process under conditions of low temperature and shortened photoperiod. Their vegetation continues with the onset of the warm period of the year, and they ear earlier than spring cultivars, which are only sown in the spring. Thus, the difference in the timing of earing of winter and spring wheat can act as one of the mechanisms of reproductive isolation in the formation of specific features of karyotypes in cultivars with an alternative sowing season. However, we are not aware of any studies of this kind.

In the present study, the potential of constitutional heterochromatin karyotyping (C-staining) in discrimination of Russian-bred common wheat varietal populations is determined. The relationship between the classification obtained, the breeding status (landraces and modern), and the growth habit (winter and spring) is analysed. Two approaches to the extraction of diagnostic features are tested. The first approach is based on the qualitative assessment of karyograms by the distribution of heterochromatic C-bands on chromosomes. We also assumed that the quantitative assessment of karyograms by the size of individual C-bands would make the classification of cultivars more adequate. The advantages and disadvantages of karyosystematics of varietal populations are discussed in comparison with genotyping based on single nucleotide polymorphism (SNP genotyping).

## Materials and methods

Plant material. Karyological analysis was performed on 87 cultivars of common wheat from different regions of Russia. The samples were 44 landraces (bred from landraces and received in the VIR collection or zoned in the USSR before 1940) and 43 modern cultivars (obtained after 1940 with hybridization) containing equal proportions of spring and winter samples (Supplementary Materials, Table S1)1.


Supplementary Materials are available in the online version of the paper: https://vavilov.elpub.ru/jour/manager/files/Suppl_Muterko_Engl_29_6.pdfhttps://vavilov.elpub.ru/jour/manager/files/Suppl_Muterko_Engl_29_6.pdf


Karyological analysis. The standard C-banding technique was used to obtain and stain chromosome preparations (Badaeva et al., 1994). Chromosomes were classified according to the genetic nomenclature (Gill et al., 1991). Karyograms were evaluated using two approaches. Qualitative evaluation was based on discrimination of chromosome types by the presence of C-bands indicated on the reference karyogram. Quantitative evaluation involved visual determination of the size of C-bands on a six-point scale (from 0 to 5) in accordance with the previously proposed recommendations (Badaeva et al., 1990). For this purpose, the chromosomes of a specific variety were compared with the generalized species ideogram and each identified band was assigned a number from 1 to 5 depending on its size: 1 – small, 2 – small but clearly visible, 3 – medium, 4 – large, 5 – very large. If a band was missing in any position, it was assigned the value “0”. Constant bands that did not vary in size were excluded from the analysis

Data analysis. Cluster analysis was performed using hierarchical and K-means clustering on Euclidean distances calculated from the initial binary data. K-means clustering was performed on a standardized (centered and scaled) distance matrix. Gap statistics (function fviz_nbclust, R package factoextra) were used to determine the optimal number of clusters for initializing the K-means algorithm. Visualization of clusters on a plane was performed using the method of multidimensional scaling of the initial binary matrix (function cmdscale, R package stats) (Becker et al., 1988).

The choice of the agglomerative hierarchical clustering method was made among eight algorithms implemented in the hclust function (R package stats) using the following approaches. 1. The empirical evaluation of the method was based on its ability to separate cultivars by the considered characteristics (breeding status, growth habit and their combination). Since the largest number of characteristic states is four (four variants of combinations of the breeding status with the growth habit), the tested topology was divided into four clusters with a volume of at least three cultivars. In each cluster, the number of cultivars with the same type of the characteristic was counted and the largest was selected, then these values were summed up for all clusters. 2. To assess the parsimony of the cladogram, expressed by the smallest number of changes explaining its topology, the Fitch algorithm implemented in the parsimony function from the R package phangorn was used (Schliep, 2011).

Cladograms were calculated with the hclust function and visualized using the R package dendextend (Galili, 2015). The assessment of clade conservation on the dendrogram, support for their monophyly, was carried out using the bootstrap method (Felsenstein, 1985) with 1,000 iterations, implemented in the boot.phylo function from the R package ape (Paradis, Schliep, 2019). Topology entanglement was calculated using the untangle function (R package dendextend) in three rounds using the random method and an additional round using the step2side method. The topological distance between dendrograms (symmetric difference) (Robinson, Foulds, 1987) was calculated with the dist.dendlist function (R package dendextend).

The selection of groups of cultivars from the cladogram was carried out by cutting its topology to a given number of clusters using the cutree function from the R package stats (Becker et al., 1988), the centers of which were calculated for each variety from the distance matrix as the average value of the distances between the given variety and the cultivars from the current cluster. Standardized coordinates of the cluster centers were used in the factor analysis

Factor analysis was performed using the principal component analysis, using the characteristics being tested as additional qualitative variables (PCA function, R package FactoMineR) (Husson et al., 2010). Pearson correlation coefficients (r) between additional variables (tested characteristics) and dimensions were calculated as square roots of eta2, and correlations between dimensions and values of these variables as square roots of cos2. Assuming a normal distribution of correlation coefficients (according to the central limit theorem), the asymptotic p-values for r > 0.40 with 87 observations were <0.01 (power = 0.9).

Multiple correspondence analysis was performed using the MCA function (R package FactoMineR) on the original binary data transformed into logical levels. The characteristics being tested were utilized as additional variables

The statistical significance of overrepresentation assumptions was assessed using Fisher’s exact test (function fisher. test from the R package stats), followed by filtering the results by p-value ≥ 0.05

Logistic regression was calculated using the glm function (R package stats), using a quasi-binomial model with link function logit

Primary data on SNP genotyping of 20 cultivars from the tested sample were obtained from the work of D.A. Afonnikov et al. (2024).

## Results

The results of karyological differentiation of 87 Russian cultivars as well as the analysis of the conjugacy of their breeding status and growth habit with the resulting classification are consistently presented according to two types of diagnostic features: chromosome type and C-band size

**Qualitative assessment of karyograms. Chromosome type as a differentiating feature**

*Association of the chromosome type with the breeding status and the growth habit of cultivars*

Using chromosome type as a diagnostic feature, a total of 205 unique karyograms were detected for chromosomes from the A, B, and D genomes (Fig. S1). Chromosomes 4D and 5D were excluded from the analysis, since the C-staining method used did not allow for reliable separation of their polymorphic variants. Although the frequency of occurrence of individual chromosome types varied greatly depending on the breeding status of cultivars, their growth habit, as well as the combinations of these characteristics (Fig. S2), no differences in their distribution by the characteristics tested were found between the genomes and groups of homoeologous chromosomes; in the groups of cultivars with alternative forms of the tested characteristics, an almost equal number of chromosome types from each genome or homoeologous group is presented, which indicates the absence of bias in the original data. 12 groups of associated chromosome types were found, characterized by identical sets in all cultivars (Table S2). In most cases, the groups were presented in pairs, three groups contained three associated types, and one four.

**Fig. 1.**
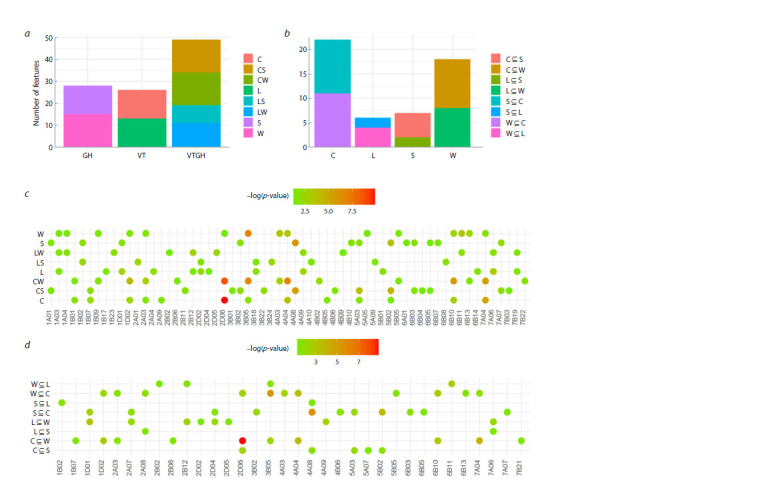
Association of the chromosome type with the breeding status and the growth habit of cultivars The number of chromosome types associated with the breeding status (VT), growth habit (GH) and combination of these characteristics (VTGH) in the total sample (a) and among winter (W) and spring (S) landraces (W⊆L, S⊆L) and modern (W⊆C, S⊆C) cultivars, and vice versa among landraces (L) and modern cultivars (C) of spring (L⊆S, C⊆S) and winter (L⊆W, C⊆W) wheat (b). p < 0.05 test values for overrepresentation in the sample of cultivars homogeneous in the tested trait from the total population (c) and subsamples (d).

To assess the statistical significance of the association of chromosome types with tested characteristics, Fisher’s exact test for overrepresentation was used in a sample of cultivars homogeneous for the trait being tested (Table S3). Most chromosome types (144, 70 %) were not informative, since the test for their overrepresentation resulted in a significance level of the alternative hypothesis p ≥ 0.05. The number of overrepresented chromosome types in modern cultivars was more than that in landraces of identical growth habit, in the absence of differences between contrasting samples in terms of the tested characteristics (Fig. 1a, c).

Similar results were obtained when assessing the overrepresentation of chromosome types among winter and spring cultivars with identical breeding status, and, conversely, among landraces and modern cultivars of identical growth habit (Fig. 1b, d). It follows that the homogeneity of modern cultivars is higher than that of landraces, and that of winter cultivars is higher than that of spring cultivars

*Cluster analysis of cultivars by chromosome type*

Cluster analysis was carried out using hierarchical and K-means clustering in order to select an approach that provides the most adequately interpretable idea of the differentiation of cultivars according to the tested characteristics

**Fig. 2.**
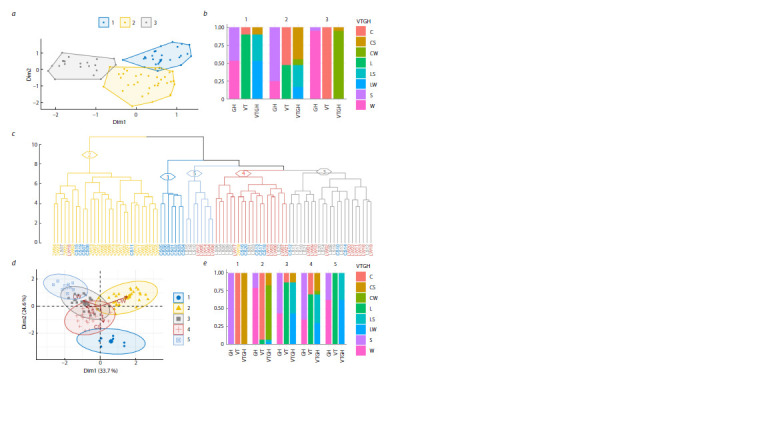
Cluster analysis of cultivars by chromosome type. a – visualization of clusters calculated by the K-means on the plane of principal coordinates; b – proportions of cultivars (W – winter, S – spring, L – landraces, C – modern) with alternative forms of the tested characteristics in these clusters; c – dendrogram of hierarchical clustering of cultivars calculated by the Ward method. For clarity, the first five clades with their representation on the plane of principal components (d) and the corresponding proportions of cultivars with alternative forms of the tested characteristics (e) are highlighted in color.

According to gap statistics, the optimal number of clusters for K-means algorithm is three. Although visualization of the clusters on the principal coordinate plane confirmed good resolution (Fig. 2a), only the third cluster was highly homogenous in terms of the characteristics under consideration (Fig. 2b). It included 21 exclusively modern cultivars, predominantly of the winter growth habit (20 cultivars). The first cluster contained 30 cultivars, with a predominance of landraces (27 cultivars), equally represented by the growth habit (16 winter and 14 spring). The remaining cluster of 36 cultivars was the most heterogeneous (Fig. 2b).

It is important to note that, based on these results, the cultivars were optimally divided into three clusters, rather than the expected four, assumed by the number of contrasting forms of tested characteristics (landraces/modern, winter/ spring, or their combinations) considered, and modern cultivars were divided by the growth habit, with the majority of winter cultivars (87 %) being allocated, while landraces were not distinguishable by this trait. At the same time, CS were grouped with landraces, and were mostly concentrated in the cluster with a predominance of spring landraces (cluster 2).

The choice of the agglomerative hierarchical clustering method was made empirically, according to the best separation of cultivars by the considered characteristics, as well as by the least number of changes explaining the tested topology (Table S4). Both approaches predict the best result for the cladogram topology calculated by the Ward method, taking into account the clustering criterion (method “ward.D2”).

In the dendrogram of cultivars, two major clades, characterized by the greatest distance from each other, divide modern winter cultivars (22 of 23) and the remaining groups (Fig. 2c). More than 70 % of the dispersion in the average distances between cultivars and major clades is explained by this division, namely, the allocation of CW into a separate cluster. With further pruning of the topology, a small group of seven CS and a large cluster uniting almost all landraces (42 of 44) are identified. The completely homogeneous cluster CS (cluster 1), although characterized by a much greater distance from both landraces (cluster 3) and modern winter cultivars (cluster 2) than the distance of the latter two from each other, is more strongly attracted to the group of cultivars of predominantly landraces (the total length of branches between clusters 1 and 3 is 2.4 times shorter than that between clusters 1 and 2), among which there are already 40 % of CS (Fig. 2e). This tendency is more clearly reflected in the plane of the main factors of attraction of cultivars to clusters (Fig. 2d). Here, the first component is positively correlated with the inclusion of cultivars in cluster 2 (r = 0.85), and the second is negatively correlated with the inclusion of cultivars in cluster 1 (r = –0.94). In the principal component plot, most of the CS (75 %) are localized in the negative coordinates of the first dimension. CS are highly correlated with the second dimension (r = 0.98), the correlation with which is also more pronounced for LW (r = 0.63) and LS (r = 0.31) than for CW (r = 0.13). Thus, the major clade from clusters 1 and 3 unites most CS with landraces. It is noteworthy that in cluster 3, CS are grouped with landraces of predominantly spring type, suggesting that modern spring cultivars have undergone less intensive breeding, retaining a greater similarity of karyograms with landraces. Despite the similarity of results between alternative cluster analysis approaches, hierarchical clustering provides a more adequately interpretable representation of variety differentiation. Support for monophyly of most clades was low, but statistically significant (9.3–99.3 %) for all top-ranking clades (Fig. S3).

Optimization of the branch length (cophenetic distances between cultivars) of the current dendrogram topology by the likelihood maximization method resulted in compression of clusters and their better resolution on the plane of principal coordinates (Fig. S4). The logarithm of likelihood (logLik) increases from –12,355 to –4,115. The separation of cultivars by breeding status becomes more convincing, but still imperfect, whereas landraces completely merge by the growth habit, but the splitting of modern cultivars by this characteristic increases.

*Correspondence analysis of cultivars by chromosome type*

**Fig. 3.**
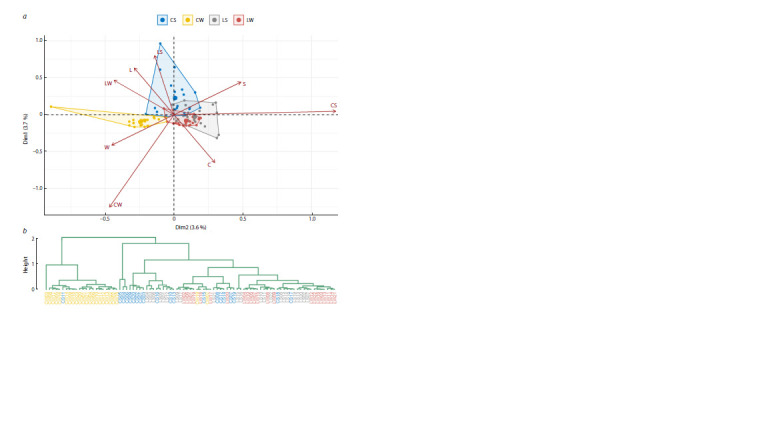
Correspondence analysis of cultivars by chromosome type. a – distribution of cultivars (W – winter, S – spring, L – landraces, C – modern) on the plane of the first two principal dimensions; b – dendrogram calculated from the coordinates of cultivars on the plane of principal dimensions.

In Figure 3a, the distribution of cultivars is presented on the plane of the first two dimensions. The proportion of explainable variance is evenly distributed between the main dimensions (3.7 and 3.6 %, respectively). However, the purpose of the present correspondence analysis is not to assess the contribution of individual chromosome types to the distribution of cultivars, but to study the relationship between this distribution and the tested characteristics, represented here by additional qualitative variables, based on their correlations with the dimensions

The first dimension is highly correlated with the characteristic combining the breeding status and the growth habit of cultivars (r = 0.66), and to a greater extent, with their growth habit (r = 0.47) than with the breeding status (r = 0.29, p > 0.14). The characteristic combining the breeding status and the growth habit of cultivars is also best correlated with the second dimension (r = 0.80), but, unlike the first dimension, mainly due to the breeding status (r = 0.64), rather than the growth habit (r = 0.43). Thus, while the growth habit correlates equally with both dimensions, the correlations of the breeding status of cultivars with the main dimensions differ by more than two times. In addition, the characteristic combining the breeding status and the growth habit also tends more towards dividing cultivars by breeding status (the correlation with the second dimension is 21 % higher than with the first), suggesting that the separation of cultivars is more likely to be determined by their breeding status than by the growth habit. It is characteristic that the tendency to group by breeding status is most pronounced for CW (r = 0.75, with the second dimension) and LS (r = 0.47, with the second dimension), but not for CS (r = 0.03 [p > 0.89], with the second dimension). The correlations of CW and LS with the growth habit are so small (r = 0.28 and r = 0.08, with the first dimension) that they are statistically insignificant (p > 0.17 and 0.87), in contrast to CS (r = 0.64 [p < 1e–4], with the first dimension). Landrace winter cultivars (LW) equally weakly and statistically insignificantly correlate with both dimensions (r = 0.27 and 0.25 [p > 0.20] with the second and first dimensions, respectively). Thus, the splitting of the sample is detected only among modern cultivars, but not landraces. In this case, the allocation of CW is mainly due to their breeding status, which does not, however, affect the distribution of CS.

On the plane of the main dimensions, it is also possible to see how much modern cultivars differ in the growth habit, the areas of clusters of winter and spring samples not overlapping, and how tightly the landraces are merged according to the same characteristic. With the exception of CW03, CW are the most homogeneous (CB = 0.42), they form a compact group in which a dense core of seven cultivars can be distinguished (CW02, CW09, CW14, CW15, CW18, CW21, and CW23). Landraces are also grouped relatively compactly (CB = 0.89), while CS form the most diffuse cluster (CB = 1.02), and therefore, are the most heterogeneous.

In view of the discovery of new factors that reveal hidden trends in the distribution of cultivars, a comparative analysis of their hierarchical clustering was carried out. In this regard, the coordinates of cultivars on the plane of principal dimensions were utilized to calculate the distance matrix, and clustering of cultivars was carried out using a similar method of calculating topology as in the original data (Ward method). In the new version of the cladogram (Fig. 3b), despite the complete restructuring of the topology (topology entanglement 97 %, topological distance 166 branches), the separation of modern cultivars has significantly improved. In particular, the CW cluster became more homogeneous, landraces and all spring cultivars except CS11 were completely excluded from it, sample CW03, carrying three chromosomal introgressions from T. miguschovae, stood out, and CW07 moved to the cluster of landraces. The cluster of CS cultivars split, but they still grouped with landraces, mainly of the spring growth habit. The distances between cultivars also changed, but their correlation remained at a high level (r = 0.80, p < 1e–5).

**Quantitative assessment of karyograms. Size of C-bands as a differentiating feature**

*Association of C-bands with the breeding status and the growth habit of cultivars*

During the quantitative assessment of karyograms by the size of C-bands, 98 diagnostic categories were identified (Fig. S5). The association of the C-bands with the tested trait was assessed using logistic regression, during which the statistical significance of the increase in the chances of detecting a C-band in cultivars with a given form of the tested characteristics was calculated with an increase in its size by one unit.

**Fig. 4.**
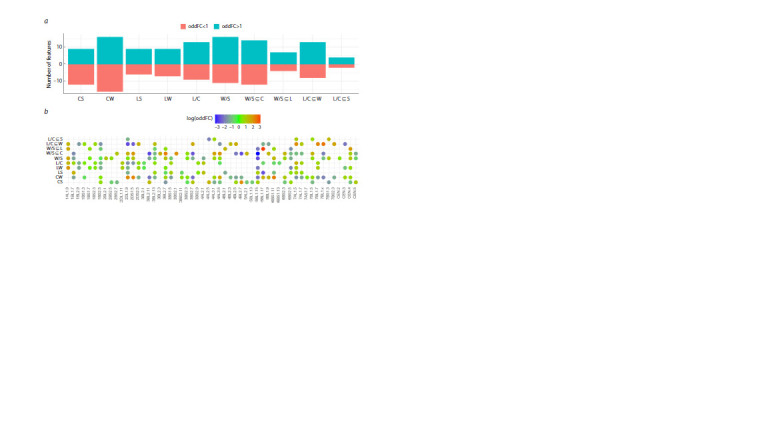
Association of C-bands with the breeding status and the growth habit. a – the number of C-bands associated with the breeding status, the growth habit and the combination of these characteristics in the total sample and in the subsamples of winter (W), spring (S), landraces (L) and modern (C) cultivars, as well as among winter and spring landraces (W⊆L, S⊆L) and modern (W⊆C, S⊆C) cultivars, and vice versa, among landraces and modern cultivars of spring (L⊆S, C⊆S) and winter (L⊆W, C⊆W) wheat; b – the multiplicative factor of the odds ratio for the C-band occurrence (oddFC) in the subset of cultivars similar by the tested characteristics from the total sample and subsamples.

When assessing the odds ratio of detecting C-bands in cultivars that differ in breeding status, statistically significant results (p < 0.05) were obtained for 22 C-bands, with the number of C-bands more clearly detected in landraces being 1.4 times greater than that in modern ones. The same analysis revealed 27 C-bands associated with the growth habit of wheat, with most of them (60 %) being identified with the winter cultivars (Table S5). Similarly, among both winter and spring cultivars, the number of C-bands associated with landraces was greater than with modern ones, and with the winter growth habit, greater than with the spring growth habit, both among modern cultivars and landraces (Fig. 4a). However, in modern cultivars, twice as many C-bands associated with alternative growth habit were identified as in landraces, and in winter cultivars, the number of C-bands associated with alternative breeding status was detected to be greater than in spring cultivars. The same was true when testing the association of C-bands with combinations of the tested characteristics (Fig. 4a).

The obtained results are fully consistent with the association of the tested characteristics with the chromosome type (Fig. 1), confirming the higher homogeneity of modern cultivars than landraces, and winter cultivars than spring cultivars also in the quantitative assessment of karyograms. However, when comparing landraces and modern cultivars regardless of their growth habit or in subsamples of its alternative form, in landraces, a high probability of detection exists for a larger number of C-bands. The above-mentioned homogeneity of the sample of modern cultivars when testing a combination of characteristics is achieved due to a larger fraction of null alleles (the probability of the absence of a C-band on a chromosome, oddFC < 1), and in the case of testing subsamples from modern cultivars and landraces, it is due to better differentiation of karyograms of modern cultivars that differ in the growth habit. Thus, not only the greater disunity of spring and winter samples in the pool of modern cultivars than in landraces is confirmed, but also the ability to identify it in the course of associative analysis during the quantitative assessment of karyograms, as opposed to the qualitative assessment of karyograms

It should be noted that the degree of association (the multiplicative factor of the odds ratio) was higher for most of the C-bands associated with CW (Fig. 4b). Thus, most of the C-bands (presence or absence) associated with modern cultivars or winter growth habit are specific for CW. Consequently, modern winter cultivars have twice as many specific karyological features (C-bands) that allow us to allocate them. In total, for all of the above-mentioned variants of analysis for the association of C-bands with the tested characteristics, less than half of them (45 %) were not informative.

*Cluster analysis of cultivars by C-band size*

**Fig. 5.**
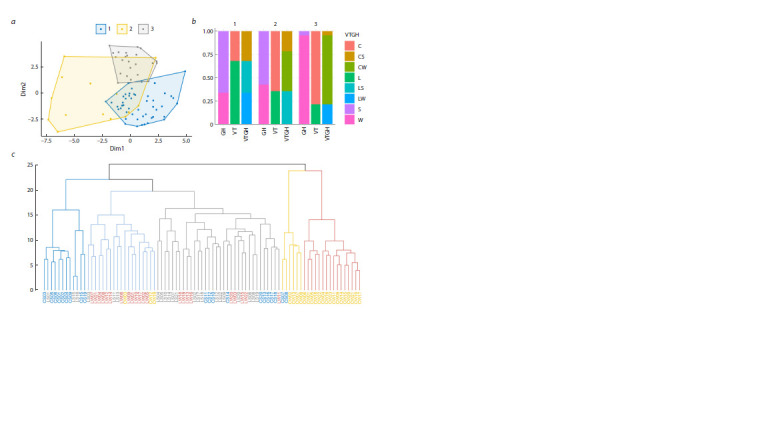
Cluster analysis of cultivars by the size of C-bands. a – visualization of clusters calculated with the K-means on the plane of principal coordinates; b – proportions of cultivars with alternative forms of the tested characteristics in these clusters (W – winter, S – spring, L – landraces, C – modern); c – dendrogram of hierarchical clustering of cultivars calculated by the Ward method (the first five clades are highlighted in color for clarity).

Although the gap statistics algorithm predicts optimal seven clusters, the best resolution is provided with three. But even in this case, only two of them are almost completely separated (Fig. 5a, b). In one of these clusters, CW predominate (74 %), with 17 of the 23 modern winter cultivars included in this cluster. The other, largest cluster contains predominantly landraces (68 %), equally represented by the growth habit. The majority of CS (80 %) also entered here, confirming their gravitation towards landraces rather than modern cultivars. The high heterogeneity of the clusters by the tested characteristics makes this clustering approach not very useful for differentiation of tested cultivars.

The results of hierarchical clustering performed by the Ward method (predicting the best separation of cultivars by the tested characteristics and the assessment of topology parsimony, Table S6) are, on the whole (in terms of separation into major clades), similar to the above classification of cultivars by chromosome type, although the topologies of the corresponding dendrograms are completely entangled (up to 90 %, topological distance of 144 branches, Fig. S6, S7), and only 13 common clades, which, however, are untangled by 62 %, retain a topological distance of 14 branches (Fig. S8). In the new version, it should be noted that the CW clade is more homogeneous and is divided into two clusters of unequal volume (Fig. 5c). The validity of this division is confirmed by the high conjugacy of the sizes of a number of C-bands on chromosomes 1BS and 3BS (Fig. S9). In addition, CS and LW are also grouped more densely and uniformly. Finally, the topology of the dendrogram is distinguished by greater support for the monophyly of the clades (Q3 = 43.5 %, Fig. S10), which follows from the better conjugacy of the diagnostic features obtained during the quantitative assessment of the karyograms

*Factor analysis by C-band size*

**Fig. 6.**
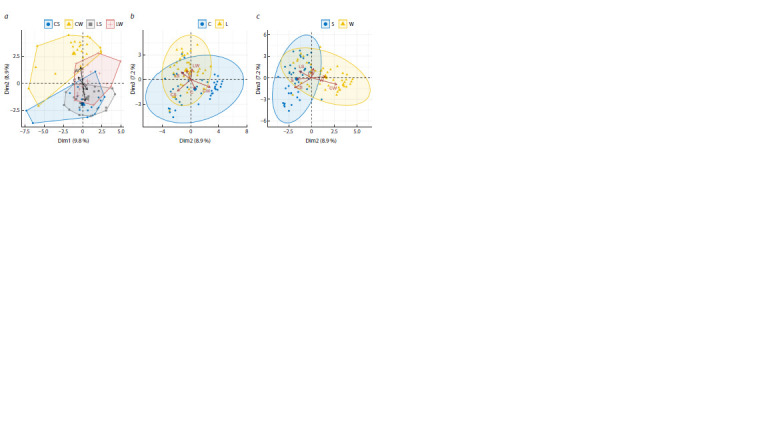
Factor analysis by C-band size. The distribution of cultivars is presented on the plane of the first two (a), as well as the second and third (b, c) principal components. In the latter case, groups of cultivars (W – winter, S – spring, L – landraces, C – modern) with identical breeding status (b) and growth habit (c) are distinguished.

Factor analysis was carried out using the principal component (PC) method, utilizing the tested characteristics as additional variables. In the plane of the first two PCs (Fig. 6a), only CW is well separated, with a dense core of eight cultivars. Modern spring cultivars almost completely overlap with landraces, mainly also spring ones. This differs significantly from the distribution of these clusters observed during the analysis by chromosome type, where clusters of spring cultivars of alternative breeding status overlapped to a much lesser extent. Another distinctive feature of the results of this analysis is the emerging trend in the division of landraces by growth habit. Finally, in connection with the transition from a binary scale to a six-point scale, the density of clusters changes, they become more sparse, the resolution in the division of cultivars increases. Proportion of explainable variance falling on each of the components is less than 10 %, which confirms the weak consistency of diagnostic features. However, their total value (18.7 %) is 2.6 times greater than that obtained from the principal dimensions during the correspondence analysis of cultivars by chromosome type.

In this analysis, however, it is not the influence of combinations of features (size of C-bands) on the discrimination of cultivars that is of interest, but the identification of trends in the grouping of cultivars by the tested characteristics, which, however, weakly and statistically insignificantly (p > 0.24) correlate with the first PC. In this regard, it makes sense to consider the distribution of cultivars in the space formed by the second and third PCs (Fig. 6b, c), which are highly correlated with the tested characteristics. In particularly, the correlation of the growth habit is maximum with the second PC (r = 0.71), while the breeding status of cultivars is more strongly correlated with the third PC (r = 0.57). Since the correlation with the growth habit is higher than with the breeding status, the separation of cultivars by this characteristic is also better (cos2 = 0.78 for winter and spring types, and cos2 = 0.52 for landrace and modern status); this is another difference from the correspondence analysis by chromosome type. Consequently, in addition to the clear allocation of CW, there is a tendency to separate cultivars by growth habit (Fig. 6c), which prevails over their breeding status (Fig. 6b), and this applies in particular to modern cultivars. CS are more strongly attracted to LS than CW to LW, as a result of which landraces are grouped more densely than modern ones. The movement of the LW cluster in the direction of the winter growth habit (W, the positive direction of the second dimension Dim2) shifts the perpendicularly directed influence of their landrace status (L, the positive direction of the third dimension Dim3); as a result, this cluster moves diagonally (Fig. 6a). It is interesting to note the influence of modern breeding on the isolation of winter wheat. On LW, the breeding status has a much stronger influence than the growth habit (cos2 = 0.32, with Dim3, and cos2 = 0.008, with Dim2), whereas in the CW cluster, the influence of the growth habit prevails (cos2 = 0.08, with Dim3, and cos2 = 0.74, with Dim2).

**Comparative analysis of karyological classification with SNP genotyping**

To assess the advantages and disadvantages of the karyological approach to classifying wheat cultivars in comparison with SNP genotyping, their resolution in differentiating a sample of 16 winter cultivars, contrasting in breeding status, and four modern spring cultivars was tested. The analysis was carried out on 120 features during assessment by chromosome type, 88 features during quantitative evaluation of karyograms by the size of C-bands, and 3,126 features during SNP genotyping; in the latter case, only unambiguously interpretable SNP markers were used, excluding heterozygotes

**Fig. 7.**
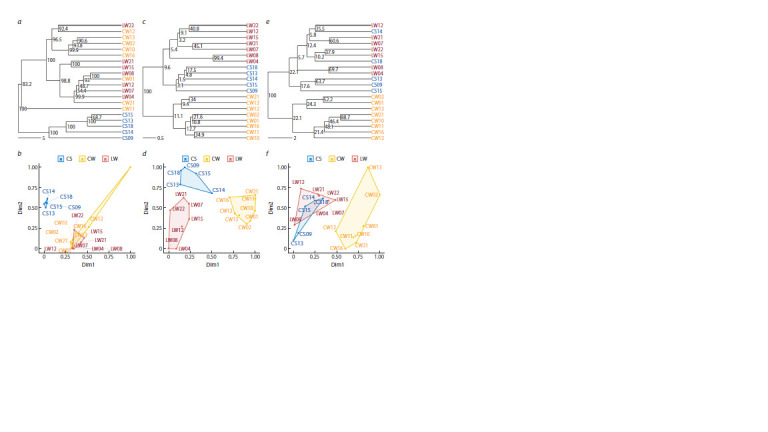
Comparative analysis of karyological classification with SNP genotyping. Dendrograms and distribution of cultivars (W – winter, S – spring, L – landraces, C – modern) on the plane of principal coordinates are presented according to the results of SNP genotyping (a and b), chromosome type analysis (c and d), and C-band size (e and f).

When comparing the qualitative method of evaluating karyograms with SNP genotyping, the correlation of distances between cultivars was very low (r = 0.22, p > 0.07), the entanglement of the topologies of the dendrograms calculated by the Ward method was no less than 40 %, with a topological distance of 32 branches. Even the minimum estimate of the preservation of monophyly of the clades calculated based on the results of SNP genotyping (48.7 %) exceeded the third quartile when using the chromosome type as a diagnostic feature (Q3 = 34.5 %) (Fig. 7a, c). On the plane of the main dimensions, all three groups of cultivars are completely separated by chromosome type (Fig. 7d). According to the results of SNP genotyping, the cultivars are well separated only by growth habit, while the winter wheat group is not distinguishable by breeding status (Fig. 7b). In this regard, the advantage of karyological analysis by chromosome type in classifying cultivars by breeding status seems obvious, due to its greater resolution at the given discrimination criteria, despite the smaller number of diagnostic features and their low consistency

The use of C-band size as a diagnostic feature led to an even greater decrease in the similarity between dendrograms, increasing the entanglement of topologies to more than 80 % and increasing the topological distance to 36 branches, with a complete loss of distance correlation. Otherwise, the differences between alternative approaches to assessing karyograms in relation to SNP genotyping are similar to the previously noted features of the quantitative method: better consistency of the original data in supporting the monophyly of the clades (Q3 = 56.4 %, Fig. 7e), allocation of CW as a more sparse cluster, and intersection of CS clusters with LW (Fig. 7f ). Thus, regardless of the approach to evaluating karyograms, the significant differences between karyological classification and SNP genotyping include the distinct separation of modern winter cultivars and the CS tendency toward landraces, which is more pronounced in the classification by C-band size.

## Discussion

The present study is aimed at assessing the potential of karyological analysis in differentiating such complex objects as varietal populations using modern cultivars and landraces as an example. In this regard, 1) two methods of obtaining diagnostic features from karyograms were tested for suitability for discrimination, based on the limits of their variability (degree of polymorphism), informativeness and resolution; 2) an assessment of the advantages and disadvantages of this approach was carried out in comparison with SNP genotyping; 3) an analysis of karyological classifications was performed for the presence of trends in the distribution of cultivars in accordance with their breeding status and growth habit, as well as an assessment of their contribution to the observed clustering and evaluation of its statistical significance.

The diagnostic features used were based on the variability of constitutive heterochromatin blocks. Two alternative approaches to its assessment were tested for resolution in discrimination of Russian common wheat cultivars by their breeding status and growth habit. The approach based on the qualitative assessment of the karyogram uses a binary scale reflecting the presence/absence of the C-band on the chromosome (distribution of C-bands along the chromosome length). The disadvantage of this approach is that chromosomes differing only in one C-band will be classified as different types, and the difference between them will be assessed in the same way as between chromosomes with significantly different karyograms. For instance, in the classification based on this method, the CW03 sample with three unique introgressions from T. miguschovae is distinguished as a separate branch on the dendrogram and is highly isolated from the other cultivars in the space of the main dimensions during the correspondence analysis

Assuming a reduction in the number of distinguished categories due to the unification of similar chromosome types, and, consequently, a more adequate classification of cultivars, a transition was made to a six-point scale for assessing karyograms by the size of individual C-bands (quantitative assessment). Indeed, when using this approach, the number of categories allocated was halved (from 205 to 98), but, on average, the resolution of cultivars increased by the same amount (Fig. S11). The latter is due to a threefold expansion of the range of the evaluation scale, which allows for a more in-depth study of the genesis of cultivars, since the higher the resolution of the method, the more common features among the classified samples it is able to detect. In the course of the principal component analysis, this led not only to an increase in the proportion of explainable variance in the karyograms, but also to the revelation of the importance of less obvious factors in the systematization of the studied material. In particular, while in the classification of cultivars by chromosome type (qualitative assessment of karyograms), the “breeding factor” is of primary importance, in the classification based on the quantitative assessment of karyograms (size of C-bands), a more significant contribution of the growth habit is found. In hierarchical clustering, the advantage of moving to a six-point scale is expressed in a denser and more homogeneous grouping of cultivars by the considered characteristics, justifying the expected increase in the adequacy of their classification. Support for the monophyly of the clades was also higher, indicating a better consistency of the initial data (conjugacy of diagnostic features). Finally, in the study of the association of alternative differentiating features with the breeding status and the growth habit of cultivars, despite the reduction of diagnostic categories, the uninformative fraction decreased from 70 to 45 %. However, despite the above-mentioned advantages of this approach, its resolution in discriminating modern spring cultivars from landraces is significantly inferior to the qualitative approach in assessing karyograms. In addition, in interpreting the results, one should accept the risks of the influence of the subjective factor when assessing the size of C-bands.

Since C-bands represent segments of inactive chromatin characterized by a high degree of evolutionary conservatism, their use as classification features is more justified in the study of the genesis of cultivars than the specificity of their genotypes. We also assume that the karyological features of this type are predominantly selection-neutral and are not subject to the adaptive action of natural selection. In this regard, any association of a karyogram and a genotype with a specific growth habit reflects only the commonality of their origin. In view of the peculiarities of intensive selection, it is likely that such associations will be more strongly expressed among modern cultivars than among landraces. Since varietal populations are locally adapted to the ecological and geographical conditions of the cultivation region, a relationship is expected between the wheat growth habit and its origin. We also hypothesized that more homogeneous modern cultivars, which were often obtained using similar sets of elite founder cultivars, would be characterized by a higher correlation of the karyogram with the growth habit than heterogeneous and locally adapted landraces. This assumption is based on the fact that genetic drift events such as the “bottleneck” and the “founder effect” occur during domestication, and modern cultivars experience the impact of these evolutionary events again when they are separated from the pool of locally cultivated wheats (Ladizinsky, 1985; Tanksley, McCouch, 1997; Feldman, Levy, 2023). For instance, a study of a sample of Chinese bread wheat cultivars based on FISH analysis revealed a significant role of the “founder effect” in their clustering (Huang et al., 2018; Hu et al., 2022). As a diagnostic feature, the authors of these works took the similarity of the distribution patterns of several polymorphic DNA probes, which corresponds to the qualitative feature “chromosome type” in our study. In addition, the more intensively the cultivars are involved in breeding, the more they diverge and become isolated, which leads to a narrowing of the genetic diversity of modern cultivars (Reif et al., 2005; Haudry et al., 2007; Sindhu, 2022). Based on the fact that the number of winter common wheat cultivars approved for use in the territory of the Russian Federation significantly exceeds the number of spring cultivars (432 and 336, respectively; State Register..., 2024), it can be assumed that the breeding of modern winter wheat is carried out more intensively, and it should be more isolated from landraces. Indeed, the factor analysis showed a strong association of the growth habit with the classification of modern cultivars by chromosome type, while the karyograms of landraces are poorly distinguishable by this characteristic. At the same time, with an increase in the resolution of the method, the association of the growth habit with chromosomal differentiation slightly increased, with a tendency to separate landraces by growth habit. Earlier, when analyzing 20 Russian cultivars using SSR and ISBP markers, a division into subclusters of winter and spring forms was also noted (Adonina et al., 2017).

The study revealed different effects of modern breeding on the isolation of winter and spring wheat. In particularly, modern winter cultivars, regardless of the type of differentiating feature (chromosome type or C-band size) and classification approach, due to the greater similarity of karyotypes, are distinguished as a strictly delimited group, in which a dense core of 7–8 cultivars with a very similar karyogram is found. Thus, the growth habit of these cultivars is not only associated with the corresponding alleles of the vernalization genes, but reflects a karyotype that is separate from spring and landrace cultivars, formed in the course of long-term and targeted breeding. This observation confirms the greater commonality in the genealogy of modern winter cultivars (Novoselskaya-Dragovich et al., 2015). This is also evidenced by their high homogeneity, according to the number of associated karyological features of both types, and intervarietal distances. Indeed, most of the CW included in the present study had the Bezostaya 1 variety in their pedigree, which could probably also have affected the isolation of this group.

Modern spring cultivars, unlike winter cultivars, are probably less intensively involved in breeding. They have experienced less influence of artificial selection and have retained more affinity with landraces, mainly also of the spring growth habit. The latter is especially clearly evident in the classification based on the quantitative assessment of karyograms where the cluster of modern spring cultivars is almost completely overlapped with landraces, while none of them are included in the cluster of modern winter cultivars (Fig. 3a, 6a). Factor analysis also emphasizes the predominance of the growth habit over the breeding status in the distribution of cultivars. It should be noted that, despite the closeness of modern spring cultivars to landraces, both approaches to karyological classification reveal a tendency in their isolation from the latter. This is manifested both in the homogeneity of the CS clustering among landraces and in the allocation of some of them into an independent clade. Considering the complete separation of CW and CS, as well as the tendency in the separation of landraces by this characteristic, the growth habit appears to be a significant factor in the differentiation of cultivated wheat, the reproductive isolation of varietal populations, and their eco-geographical distribution.

When comparing the karyological method with SNP genotyping, both its key disadvantages and advantages in classifying wheat cultivars have been identified. Among the disadvantages, the limited number of diagnostic features and their low consistency are mainly highlighted. If the quantitative limitation is due to the nature of the features of this type, then the weak support for the monophyly of clades on the dendrogram is associated not only with their low conjugacy in this sample, but also follows from the first disadvantage (Rokas, Carroll, 2005). The low consistency of karyological characters can be explained by the high frequency of C-band recombination caused by both their high disunity on the chromosome and combinatorial events in the distribution of homologous chromosomes during meiosis (Blary, Jenczewski, 2019; Koo et al., 2020; Mason, Wendel, 2020; Fan et al., 2021), as well as changes in chromosome staining patterns as a result of introgression and chromosomal rearrangements. A certain contribution is made by the technology of preparation and staining of metaphase plate preparations, as well as a considerable amount of subjectivity in assessing the size of C-bands.

Considerations of the monophyly of clades come from the systematics of species based on their phylogeny. However, the assumption of complete reproductive isolation, usually applied to species, is unacceptable for freely interbreeding wheat cultivars, and in the obtaining of cultivars, as was noted in the introductory part of the article, samples from geographically separated populations and even representatives of related taxa were often used. The assessment of the monophyly of clades in the dendrograms presented in the current study reflects mainly the degree of conjugacy of diagnostic characters. Indeed, the support for the monophyly of the clades was somewhat higher when using the quantitative karyotyping method. And although the statistical reliability of monophyly was significant for all clades of the highest rank, regardless of the approach to assessing the karyograms it sharply decreased to complete disappearance when moving to clades of lower ranks. At the same time, the justification for monophyly was incomparably stronger and statistically significant for all clades of the dendrogram calculated using SNP genotyping, in which the number of diagnostic features was an order of magnitude greater, and their conjugacy was higher, due to the higher mapping density on chromosomes

Nevertheless, the value of differentiating features in classification problems is determined by their ability to discriminate a sample by the required qualities. While SNP markers provide an idea of allelic, gene variability, polymorphism by chromosomal markers reflects the variability of the karyotype as a whole. Since the structure of heterochromatic C-bands is not subject to genetic recombination and conversion, they are evolutionarily more stable. Chromosomal aberrations, also related to chromosomal markers, lead to significant disturbances in the meiosis of heterozygotes, thereby leveling the heterogeneity of the population and contributing to the preservation of the authenticity of the original populations over time, as, for example, noted for the group of European emmer with the marker 5B-7A translocation (Dedkova et al., 2009; Badaeva et al., 2015b). Thus, chromosomal markers seem to be more reliable for systematization of wheat varietal sets from the point of view of reliability and reproducibility of classification, especially when the number of diagnostic features is limited. Combinatorial variability at the chromosome level forms new karyotypes (classification categories in karyological differentiation), but does not change the combinations of features in linkage groups (often reflected in conjugated markers in SNP genotyping). As a result, a larger number of agreed diagnostic features, in itself, does not yet mean better discrimination of their carriers, since the latter also depends on the type of these features. Indeed, comparison of SNP and chromosomal markers, in particular when using the chromosome type, but not the size of C-bands as a diagnostic feature, confirmed the superiority of the latter in discrimination of 20 selected wheat cultivars both by breeding status and by growth habit, despite their low consistency.

It is significant that the use of chromosomal markers reliably differentiates winter wheat cultivars depending on their breeding status, whereas in the case of SNP genotyping, these same cultivars are grouped together. As noted above, this circumstance may be due to the difference in the type of variability detected by SNP and chromosomal markers; in other words, karyotypes of different origin may not differ in the alleles of many genes (hence the high consistency of SNP markers). It is possible that an increase in the number of SNP markers will increase the resolution of this method in discriminating the tested sample of cultivars by breeding status. Nevertheless, the number of such markers already exceeds by more than an order of magnitude the minimum set of karyological characteristics sufficient for the successful resolution of this problem, emphasizing the advantage of the karyological method, especially with a limited number of diagnostic features

## Conclusion

The present study revealed a high potential of karyotyping by constitutive heterochromatin in discrimination of modern Russian cultivars of common wheat by the growth habit, and isolation of their winter samples from landraces. At the same time, the homogeneity of modern cultivars by the tested karyological features is higher than that of landraces, and that of winter cultivars is higher than that of spring cultivars. The resulting classification reflects the preservation of high similarity in the karyotypes of modern spring cultivars and varietal populations of landraces, as well as weak distinguishability of the karyotypes of the latter, contrasting by the growth habit. In connection with a more unambiguous association of differential chromosome staining patterns with the origin of cultivars, as well as their high evolutionary stability, it is assumed that the analysis of karyotypic variability helps to form a more accurate idea of the differentiation of wheat varietal sets according to this trait than when using SNP markers that detect gene variability, especially with a limited number of diagnostic characteristics

## Conflict of interest

The authors declare no conflict of interest.
